# Weaving Mitochondrial DNA and Y-Chromosome Variation in the Panamanian Genetic Canvas

**DOI:** 10.3390/genes12121921

**Published:** 2021-11-29

**Authors:** Nicola Rambaldi Migliore, Giulia Colombo, Marco Rosario Capodiferro, Lucia Mazzocchi, Ana Maria Chero Osorio, Alessandro Raveane, Maribel Tribaldos, Ugo Alessandro Perego, Tomás Mendizábal, Alejandro García Montón, Gianluca Lombardo, Viola Grugni, Maria Garofalo, Luca Ferretti, Cristina Cereda, Stella Gagliardi, Richard Cooke, Nicole Smith-Guzmán, Anna Olivieri, Bethany Aram, Antonio Torroni, Jorge Motta, Ornella Semino, Alessandro Achilli

**Affiliations:** 1 Department of Biology and Biotechnology “L. Spallanzani”, University of Pavia, 27100 Pavia, Italy; nicola.rambaldi01@universitadipavia.it (N.R.M.); giulia.colombo01@universitadipavia.it (G.C.); marcorosario.capodiferro01@universitadipavia.it (M.R.C.); lucia.mazzocchi01@universitadipavia.it (L.M.); anamaria.cheroosorio01@universitadipavia.it (A.M.C.O.); alessandro.raveane01@universitadipavia.it (A.R.); uperego@scciowa.edu (U.A.P.); gianluca.lombardo01@universitadipavia.it (G.L.); viola.grugni@unipv.it (V.G.); maria.garofalo@mondino.it (M.G.); luca.ferretti@unipv.it (L.F.); anna.olivieri@unipv.it (A.O.); antonio.torroni@unipv.it (A.T.); 2 Laboratory of Hematology-Oncology, European Institute of Oncology IRCCS, 20141 Milan, Italy; 3 Gorgas Memorial Institute for Health Studies, Panama City 0816-02593, Panama; maritribaldos@gmail.com (M.T.); drjmotta@gmail.com (J.M.); 4 Department of Math and Science, Southeastern Community College, West Burlington, IA 52655, USA; 5 Center for Historical, Anthropological and Cultural Research—AIP, Panama City 0816-07812, Panama; mendizabalt@si.edu; 6 Smithsonian Tropical Research Institute, Panama City 0843-03092, Panama; cooker@si.edu (R.C.); smithn@si.edu (N.S.-G.); 7 Departamento de Geografía, Historia y Filosofía, Universidad Pablo de Olavide, 41013 Seville, Spain; alejandro.garcia@eui.eu (A.G.M.); barawor@upo.es (B.A.); 8 Genomic and Post-Genomic Unit, IRCCS Mondino Foundation, 27100 Pavia, Italy; cristina.cereda@mondino.it (C.C.); stella.gagliardi@mondino.it (S.G.); 9 Sistema Nacional de Investigadores, Secretaría Nacional de Ciencia y Tecnología, Ciudad del Saber, Clayton 0816-02852, Panama

**Keywords:** Isthmus of Panama, mitochondrial DNA, Y chromosome, uniparental transmission, phylogeography, indigenous American lineages and genetic history, sex bias

## Abstract

The Isthmus of Panama was a crossroads between North and South America during the continent’s first peopling (and subsequent movements) also playing a pivotal role during European colonization and the African slave trade. Previous analyses of uniparental systems revealed significant sex biases in the genetic history of Panamanians, as testified by the high proportions of Indigenous and sub-Saharan mitochondrial DNAs (mtDNAs) and by the prevalence of Western European/northern African Y chromosomes. Those studies were conducted on the general population without considering any self-reported ethnic affiliations. Here, we compared the mtDNA and Y-chromosome lineages of a new sample collection from 431 individuals (301 males and 130 females) belonging to either the general population, mixed groups, or one of five Indigenous groups currently living in Panama. We found different proportions of paternal and maternal lineages in the Indigenous groups testifying to pre-contact demographic events and genetic inputs (some dated to Pleistocene times) that created genetic structure. Then, while the local mitochondrial gene pool was marginally involved in post-contact admixtures, the Indigenous Y chromosomes were differentially replaced, mostly by lineages of western Eurasian origin. Finally, our new estimates of the sub-Saharan contribution, on a more accurately defined general population, reduce an apparent divergence between genetic and historical data.

## 1. Introduction

The Isthmus of Panama was an obligatory passage for the first peopling of the Americas and has served as a crossroads in the movement of people and goods ever since, also playing a pivotal role during European colonization and the African slave trade [1,2,3]. According to palaeoecological and archaeological data, a human presence in Panama is attested from ≈16,000 years ago (kya) [4,5]. Agriculture incorporating domesticated plants developed 8.6 kya, and by 5.0 kya, it had strongly impacted forested landscapes in regions with intense dry seasons favoring human burning [6,7]. Around 3 kya, three Cultural Regions can be distinguished along the Isthmus: Greater Chiriquí (today the western provinces of Chiriquí and Bocas del Toro, extending into present-day Costa Rica, and the Indigenous Comarcas of the Naso Tjër Di, and the Ngäbe and Bugle), the most coherent historical unit [8], speaking Nuclear Chibchan languages [9]; Greater Coclé (today the central provinces of Coclé and Veraguas, and the Azuero Peninsula), a culturally coherent unit, even though it is not known whether it was linguistically united; Greater Darién (East Panama and Darién provinces, and four Indigenous Comarcas—three Guna and one Emberá and Wounaan). By 1500 Common Era (CE), much of Greater Darién was inhabited by people speaking the language of Cueva, which was more likely to be a lingua franca of social communication than a vernacular. It is argued that some vernaculars spoken at pre-contact villages in the Pacific watershed of today’s Darien and East Panama provinces were of Chocoan heritage [10,11,12].

This heterogeneity in culture and organization persisted upon the arrival of the Spaniards at the beginning of the 16th century CE, who described Panama’s Indigenous communities as spanning from small tribes to large chiefdoms, speaking different languages and trading all kinds of goods [1]. The arrival of Europeans had a severe impact on autochthonous populations. Many epidemics have been reported in Central America during the first century of colonization [13]. Indigenous peoples were decimated by infectious diseases—one of the main causes of the strong population decline in the Americas—such as smallpox, measles, and typhus, among others, for which Indigenous populations had no immunity [14,15]. Other factors contributing to the population decline disproportionately impacted Indigenous men, who took part in warfare and could be forced to labor in the mines and pearl fisheries [16]. The area’s high mortality and increasing demand for labor led to the importation of enslaved men and women from other American territories, including Nicaragua, Peru, and Venezuela, as well as sub-Saharan Africa. Panama became a principal redistribution point for the slave trade and mixture among African and Indigenous peoples became common in areas inhabited by Africans and their descendants [17].

Beginning in the sixteenth century, European, African, and Indigenous populations converged on the Isthmus of Panama, leading to unions among people of different geographic and ethnic origins. The ensuing relations, coerced as well as consensual, often took place between European men and women of Indigenous American and, subsequently, African origins and descent. Free and enslaved members of African, Afro-descendent, and Indigenous communities also mixed in the cities, villages, and rural areas despite royal attempts to establish distinct Spanish, Indigenous, and Black towns [18,19]. Although the early and permanent settlement of newcomers on the Isthmus of Panama following Spanish contact changed the autochthonous gene pool, perceptions vary regarding different ethnic groups’ cultural and social impact. According to the latest Panamanian census (https://inec.gob.pa/, accessed on 7 October 2021), the percentage of citizens who identify themselves as Afro-descendants is 9.2%, while 12.3% consider themselves Indigenous. The latter comprise seven groups (from west to east): Naso Tjër Di (or Naso), Bribri, Ngäbe, Bugle/Bokotà (or Bogotà), Guna, Emberá, and Wounaan. During the 20th century CE, the Ngäbe, Bugle, Guna, Emberá and Wounaan were granted territories, called “Comarcas”, administered by Indigenous authorities. At the end of 2020 CE, the Naso received their comarca (Republic of Panama, Ley 188 de 4 de diciembre de 2020, GACETA 29170—A, https://www.gacetaoficial.gob.pa/, accessed on 7 October 2021), whereas the Bribri in the Chiriqui and Bocas del Toro provinces remain unprotected by comarca status.

Recent genomic analyses of populations currently living in the Americas confirmed the existence of sex biases in the convergence of diverse ethnic groups following European contact and colonization [20,21,22]. This sex bias was often documented by the differential inheritance of uniparental lineages with three distinct geographic/population origins: Indigenous America (IAm), Western Eurasia (WEu, including also Northern Africa), and Southern (sub-Saharan) Africa (SAf) [23,24,25,26]. In Panama, the general population (individuals without self-declared ethnic affiliations living on the Isthmus for at least two generations) has shown two striking differences between the uniparental markers: (1) the high persistence of IAm mtDNA lineages and a strong impact of WEu paternally-transmitted lineages; and (2) a significant SAf contribution to modern mtDNA legacies (more than twice) with respect to the Y chromosome [27,28]. The first outcome contrasts with the demographic data of the last official census of 2010, when less than 12.3% of Panamanians identified themselves as Indigenous. Likewise, the very limited genetic input from African and Indigenous American males and their descendants detected in such uniparental studies clashes with historical evidence of the forced migration of more African men than women to the Americas from the sixteenth through the eighteenth centuries CE [17,29,30,31,32,33,34]. More recently, archaeogenomics [35] has begun to shed light on Isthmian prehistory, with surprising results. Capodiferro et al. (2021) [36] detected a previously undescribed ancestry among ancient Indigenous peoples of the Americas, which was representative of a still unsampled Late Pleistocene population of the Isthmus (UPopI) unique to this region. It was detected in ancient pre-Hispanic individuals from excavations in Panamá Viejo and adjacent Coco del Mar as well as among the self-identified members of present-day Indigenous communities and Moreno and Mestizo groups. This genome-wide analysis also provided some clues about the impact of European colonization. As expected, present-day Morenos show prevalent African ancestry (≈62%), but with a significant European contribution (≈13%), in ADMIXTURE analyses [36]. Surprisingly, Mestizos present a larger fraction of Indigenous (≈44%) than African (≈32%) and European (≈13%) genomic components and traces of East Asian variants (≈11%). As for the current Indigenous groups, Guna and Ngäbe preserved almost entirely an Indigenous gene pool (>99%), while present-day Bribri derive a fraction of their genome from European (≈13%) and African (≈6%) sources. A common feature of most indigenous groups was the population decline that probably started before contact, earlier in the Guna group, and intensified with the conquest. These findings inspired the present research to re-evaluate the modern Panamanians’ uniparental gene pool in light of the DNA donors’ self-declared affiliations.

## 2. Materials and Methods

### 2.1. DNA Sample Collection

A total of 475 healthy individuals were enrolled in this study, during a sampling campaign conducted in Panama starting in 2016. The biological specimens (saliva) were collected through mouthwash rinsing, using the saliva DNA isolation kit from Norgen Biotek. In addition to their informed consent, all participants were asked to provide genealogical information (for at least two generations) as well as ethnic affiliation and spoken language. These data were used to identify two generations Panamanian subjects unrelated on the maternal and paternal sides and belonging to different population groups. The final group of 431 unrelated individuals sampled (Supplementary Table S1) encompassed 130 females and 301 males; 200 declared themselves as part of one of five different Indigenous groups (Bribri, Naso, Ngäbe, Guna, and Emberá), while 21 individuals identified themselves as either Mestizo (*N* = 10) or Moreno (*N* = 11). These groups include mostly individuals of mixed Hispano-Indigenous and African ancestries, respectively. Finally, a total of 210 Panamanians did not self-identify as affiliated with any of the ethnic categories listed above and were grouped together as representative of the general population for the purposes of the present study. Genomic DNA was extracted from saliva samples and purified either following a standard phenol/chloroform protocol or with automated extraction performed with the Maxwell^®^ RSC Instrument from Promega Italia srl (Milan, Italy) according to the manufacturer’s protocol.

### 2.2. Mitochondrial DNA Sequencing and Haplogroup Classification

Even if a deeper classification into sub-haplogroups could be achieved through a massive sequencing of entire mitogenomes and non-recombining regions of the Y chromosome, the main goal of the present work was to compare the different sources of uniparental haplogroups. To this aim, the Y-chromosome classification was achieved through a hierarchical screening of biallelic markers, while Sanger sequencing of the mtDNA control region allowed identifying informative SNPs for a correct mitochondrial classification, as in previous studies on the general population of Panama [27,28].

The mtDNA control region was amplified through standard PCR for 475 individuals, using the following primers: nucleotide position (np) 15877 forward (5′-CAAATGGGCCTGTCCTTGTA-3′) and np 727 reverse (5′-AGGGTGAACTCACTGGAACG-3′). A segment of the control region (nps 16024–250) was sequenced at the BMR Genomics (https://www.bmr-genomics.it/) through Sanger sequencing using the Brilliant Dye terminator 1.1 kit. The sequencing primers included a forward primer (np 15973, 5′-AACTCCACCATTAGCACCCA-3′) for all individuals and a reverse primer (np 305, 5′-GGGTTTGGTGGAAATTTTT-3′) for those harboring the transition T16189C, which results in a poly-C tail causing premature termination of the sequencing reaction.

Electropherograms were aligned to the mitochondrial reference sequence (rCRS) [37] using Sequencher v4.9 (http://www.genecodes.com/), which was also used to manually determine haplotypes. Haplogroup classification was obtained with HaploGrep2 [38]. Most of the B2 sequences were initially classified as B4 due to the lack of distinguishing B2 marker positions in the analyzed segment of the control region. This makes it difficult to distinguish between the two lineages [39,40]. This issue was addressed by classifying the B4 sequences as B2 based on the complete mitogenome information for some of them, which is already available in [36].

Of the 475 initially sequenced individuals, only 431 (Supplementary Table S1) were included in subsequent mtDNA analyses. Forty-four were excluded either because a detailed assessment of the available genealogical records revealed that they were maternally related (*N* = 39) or due to the poor quality of the sequencing data (*N* = 5).

### 2.3. Y-Chromosome Genotyping and Haplogroup Classification

A total of 301 unrelated Panamanian males were sampled. Of these, 248 were successfully classified into Y-chromosome haplogroups (Table S1), including 43 male subjects genotyped with the Affymetrix Human origin 600K chip and previously analyzed in [36]. The Y-chromosome haplogroup classification was obtained by hierarchical order analysis of 54 biallelic markers of the male-specific region of Y chromosome (MSY), following the latest Y-chromosome phylogeny (https://www.yfull.com/tree/; https://isogg.org/, both accessed on 7 October 2021) and according to [41].

### 2.4. Data Analyses

The analysis of mtDNA molecular diversity indices was performed with DnaSp v.6 [42]. Heterogeneity was computed using the standard method of Nei [43].

Haplogroup frequencies and distributions for both genetic systems were compared for statistically significant differences with either the Chi-square test of independence or the Fisher’s exact test of independence, which were computed using R [44] and the XLSTAT add-on for Excel.

Uniparental genetic pairwise distances between individuals were computed using MEGA X [45], as the proportion of nucleotides at which two sequences are different (*p-distance* method). Distances were calculated on both mtDNA (*N* = 248) and Y-chromosome (*N* = 248) data, using only variable sites (disregarding indels) from the sequenced control-region portion and from the tested SNPs, respectively. We calculated intra-population distances for each system by keeping only pairwise comparisons of individuals belonging to the same population group. Conversely, we used all pairwise values to compute the mean of Y-chromosome and mtDNA distances and convert it into a dissimilarity matrix, using the R functions *mean* and *xtabs* [42]. The resulting matrix was used to perform a multidimensional scaling (MDS) using the R function *cmdscale* [44].

Principal Component Analysis (PCA), on both mtDNA and Y-chromosome haplogroup frequencies, was computed using *prcomp* from the *stats* R package [42] with the center and scale arguments set as true. Correspondence Analysis (CA) was performed on the same haplogroup frequencies using the *CA* function from the *FactoMineR* R package [46], as in [47]. All plots were generated using the R packages *ggplot2* [48] and *factoextra* (https://github.com/kassambara/factoextra).

## 3. Results

### 3.1. MtDNA

**Figure 1:**
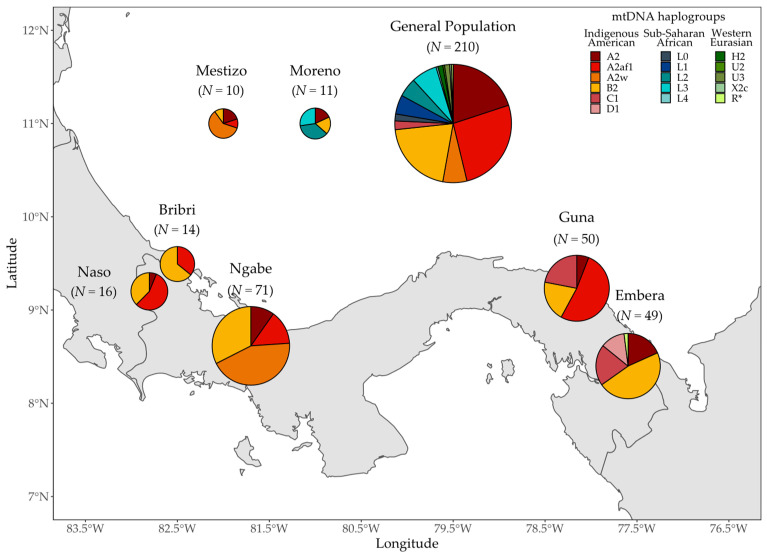
Map showing the haplogroup distribution of the 431 mtDNAs according to the self-reported affiliation of the participants in the study. Location of Indigenous groups corresponds to their specific Indigenous Comarca (Naso Tjër Di, Ngäbe, Guna, and Emberá). As for the Bribri, most of them live within Costa Rican borders, and about 3000 are settled in Panama. The size of each pie chart is proportional to the number of individuals from each group. Haplogroup origins are indicated by shades of different colors. Diereses and accents were not considered when labeling the different groups.

We generated mtDNA control-region data for 431 maternally unrelated individuals, which were sampled among the general population (*N* = 210) as well as five Panamanian Indigenous groups (*N* = 200): Naso, Bribri, Ngäbe, Guna, and Emberá (Figure 1; Supplementary Table S1). The two admixed groups, Mestizo (*N* = 10) and Moreno (*N* = 11), were represented in the final dataset.

The analyzed sequences covered nps 16024–250, thus including the complete hypervariable segment I (HVS-I, nps 16024–16383) and part of the HVS-II (nps 57–372), for a total of 796 sites, of which 161 are polymorphic. The total dataset includes 173 distinct haplotypes (haplotype diversity, Hd = 0.971; Supplementary Table S2. High-diversity values are found in the general population (Hd = 0.988) and in the Moreno group (Hd = 0.945), which also show lower proportions of IAm haplogroups. Among Indigenous populations, the highest Hd value is observed in the Emberá (0.971), whereas Naso and Bribri are characterized by the lowest diversity (Hd = 0.575 and 0.538, respectively).

The classification into mitochondrial haplogroups (Figure 1; Supplementary Table S2) revealed a prevalence of the Indigenous pan-American founding lineages (A2, B2, C1, D1), totaling 86.3% of the entire dataset and 75.7% of the general population, which is in agreement with [27]. All individuals from IAm populations belonged to Indigenous lineages, except for one Emberá whose mtDNA was identified as haplogroup R*.

The macro-haplogroup A2 is the most represented, accounting for 51.7% of all individuals, which was followed by B2 (27.1%) and C1 (6.0%). D1 is found with the lowest frequency (1.4%) and only in the Emberá group. Non-Indigenous lineages account for 13.7% of the total, with West Eurasian haplogroups representing 2.3% and SAf haplogroups representing 11.4%. A2 could be further classified into two main sub-lineages. A2w represents 11.8% of the entire dataset and is found among the Ngäbe (43.7%), the Mestizo (60.0%), and the general population (6.7%). A2af1 (24.6% of the total) is found across all Indigenous groups (except for the Emberá), even though its proportions within each particular Indigenous group are significantly different (Chi-squared test, *p*-value < 0.01) and reach the highest frequencies in Guna (52.0%) and Naso (56.3%) populations. This lineage is found in one Mestizo individual and is also the most represented in the general population (26.2%).

The haplogroup B2 is found among all Panamanian populations sampled, with significant differences between the Indigenous groups (Chi-square test, *p*-value < 0.01). The highest frequencies are reached in Emberá (46.9%) and Bribri (64.3%) populations. The haplogroups C1 and D1 are almost exclusively found in the eastern populations (Guna and Emberá), with D1 present only in the latter and C1 equally distributed (Chi-square test, *p*-value = 0.83) in both groups (≈20–22% in each population).

The Western Eurasian haplogroups (H2, U2, U3, X2c, R*) are only found in the general population (4.3%) with the exception of the previously cited R* mtDNA found in one Emberá. The Mestizo individuals do not present any European mtDNA lineage, showing conservation of Indigenous ancestries with no European contribution from a matrilineal point of view. Conversely, sub-Saharan African haplogroups are found in the general population (20.0%) and in the Moreno group (63.7%). In the latter group, showing a greater presence of mixed ancestries along the maternal line, the sub-Saharan maternal ancestries are represented by L2 (36.4%) and L3 (27.3%) lineages.

### 3.2. Y Chromosome

**Figure 2:**
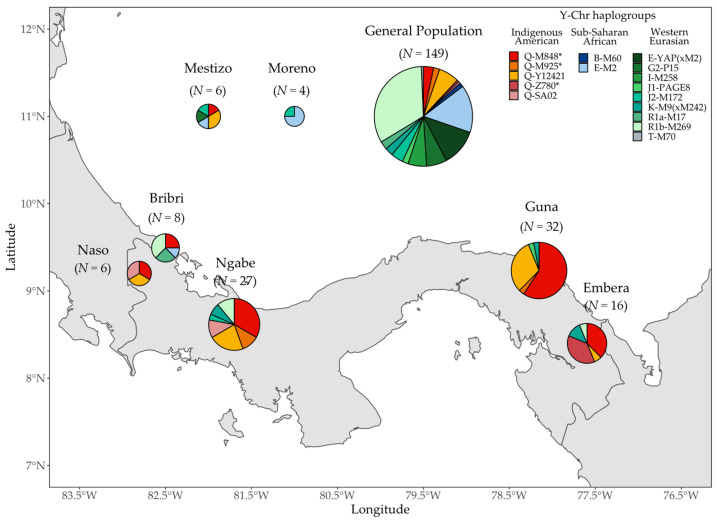
Map showing the haplogroup distribution of the 248 Y chromosomes according to the self-reported affiliation of the participants in the study (see Figure 1 for further details).

A total of 248 Panamanian males were successfully classified into 25 Y-chromosome haplogroups, with three main geographic origins: Indigenous America (haplogroup Q), sub-Saharan Africa (haplogroups B-M60 and E-M2), and Western Eurasia/North Africa (the remaining haplogroups) (Figure 2; Supplementary Table S4).

In agreement with previous observations [41,49], the Indigenous component is represented by haplogroups Q-M848 and Q-Z780. However, each of these haplogroups shows only one of its known sub-clades. For Q-M848, only the Q-M925 branch is observed, in which ≈82% of individuals belong to its sub-branch Q-Y12421, which has been so far reported in Panama, Mexico, and in the southwestern United States [41]. The remaining Q-M848 Y chromosomes are negative for the signature markers (CTS4000, Z5908, M925, CTS2731, and Y780) of its major downstream clades, and therefore, they are classified as Q-M848* (paragroup). As for Q-Z780, about 50% of the Y chromosomes belong to the Q-SA02, which is a clade that has been previously observed in few subjects from Costa Rica and Panama [41], and from Coyaima groups of Colombia [50]. The remaining Q-Z780 chromosomes (negative for SA02 and Z781) are reported as Q-Z780*.

As shown in Figure 2, the Y-chromosome lineages of different geographic origins (IAm, SAf, and WEu) are distributed quite unevenly among the populations. In the general population sample, most (69.8%) of the Y chromosomes fall within Western Eurasian haplogroups, namely I-M258, I1-M253, K-M9(xM242), R1a-M17, R1b-M269, R1b-U106, R1b-S116, G2-M547, and G2-P15 [51,52,53,54]; the South/West-Eurasian J2-M172 (xPage55) and J2-PAGE55 [55,56]; the Mediterranean (including North and East African) E-M123, E-M78, E-M81, J1-PAGE08, and T-M70 [55,57,58]. Amongst these haplogroups, the Iberian R1b-S116 [59] stands out, being by far the most frequent lineage in the general population (28.2%). The second most frequent source is represented by African haplogroups (16.8%), with the West sub-Saharan African E-M2 [60] being second in frequency (15.4%) after the Iberian R1b-S116, and the South sub-Saharan African B-M60 [61,62] represented only by two individuals. Lastly, the least represented source refers to the Indigenous American haplogroup Q sub-lineages, accounting for 13.4%, mainly represented by the Isthmo-Colombian Q-Y12421 (6.7%) [41], whose frequency is not very distant from that of all the remaining IAm haplogroups.

Among the admixed groups, Mestizo and Moreno present different genetic compositions reflecting their respective origins. Mestizo accounts for, in order of frequency, Indigenous American, Western Eurasian, and African contributions, while amongst the four Moreno Y chromosomes, three were E-M2 and one was J2-M172, thus identifying a predominantly African paternal ancestry (E-M2).

Most individuals who self-declared Indigenous affiliations have retained an IAm paternal ancestry (80.9%). Only the Bribri differ from this trend, as six out of the eight males analyzed harbored Y chromosomes not belonging to haplogroup Q (five of European ancestry and one from Africa). The only two haplogroup Q Y chromosomes (25.0%) found in the Bribri both belong to Q-M848*. The frequency of this paragroup ranges from 33.3% in the Ngäbe and the Naso to 59.4% in the Guna. The most frequent sub-haplogroup among Indigenous groups is the Isthmo-Colombian Q-Y12421, which reaches its highest frequencies (≈31%) in the Guna and the Naso. Other clades highlight a number of differences between IAm populations: (i) the Q-Z780 sub-haplogroup Q-SA02 is only observed in western groups (Naso and Ngäbe), while all the Q-Z780 remain classified as Q-Z780* in the Emberá (from the eastern part of the country); (ii) no Q-Z780 Y chromosomes were identified in the Guna; (iii) Q-M925* is almost exclusively found in the Ngäbe with the exception of one Guna.

Heterogeneity values were calculated for each group considering both the totality of the observed haplogroups (H_tot_) and the Indigenous haplogroups only (H_HgQ_). H_tot_ values reveal that Indigenous groups are almost as heterogeneous (values between 0.742 and 0.893) as the admixed general population (H = 0.883), with the only exception of the Guna (H = 0.565). On the other hand, H_HgQ_ values show that, apart from external contributions, a certain diversity of the IAm component is maintained in all Indigenous groups.

### 3.3. Combining MtDNA and Y-Chromosome Results

To compare Y-chromosome and mtDNA data, we first restricted the total mtDNA dataset of 431 individuals to the one used for Y-chromosome analyses (248 males unrelated on both the paternal and maternal sides). We did not find any statistically significant difference when comparing the mtDNA haplogroup distribution between the two datasets (Fisher’s exact test, *p*-value = 0.998). The mtDNA haplogroup frequencies for this dataset are summarized in Table S3 and Supplementary Figure S1.

**Figure 3:**
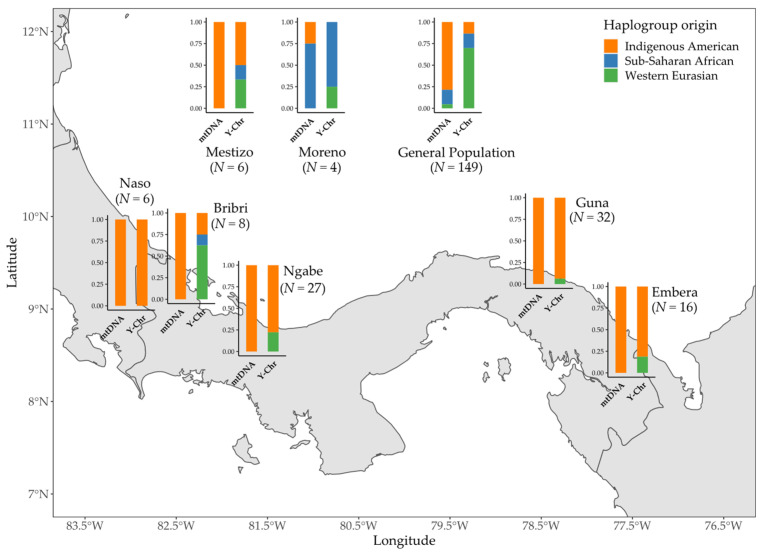
Map showing the distributions of mtDNA and Y-chromosome haplogroup origins (indicated by different colors) among 248 Panamanian males.

The proportion of Indigenous lineages is higher for the mtDNA than for the Y chromosome in all Indigenous groups, except for Naso, as well as in the general population (Figure 3). In particular, Panamanian Indigenous groups reach a frequency of 100% of Indigenous lineages when looking at the maternal side, but with an important WEu contribution for the paternal side. This contribution is predominant in the Bribri, who also show one individual belonging to the sub-Saharan Y-chromosome lineage E-M2. As expected, the Moreno group shows the lowest proportion of IAm lineages for both systems, since most of the individuals bear SAf haplogroups. The admixed Mestizo and the general population groups are characterized by similar patterns, having predominant IAm ancestries for the mitochondrial DNA and substantial WEu and SAf Indigenous contributions for the Y chromosome. Here, in contrast to previous estimates [27,28], the general population shows the same proportion of SAf lineages (16.8%) for both uniparental systems.

**Figure 4:**
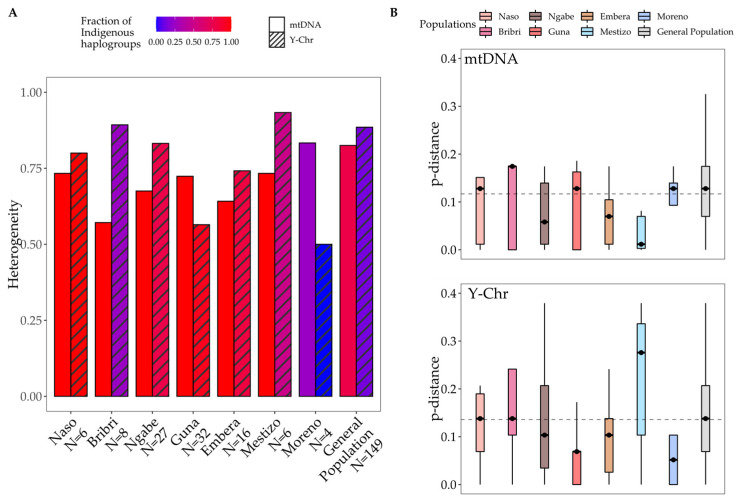
Heterogeneity based on haplogroup frequencies (**A**) and intra-population uniparental genetic pairwise distances (**B**) of different Panamanian groups computed for mtDNA and the Y-chromosome. In panel A, heterogeneity bars are shaded (from blue to red) according to the proportion of Indigenous lineages in each population. In panel B, dashed lines represent the mean value of all distances for mtDNA (above, mean = 0.12) and for the Y-chromosome (below, mean = 0.14).

Genetic diversities, computed as heterogeneity based on haplogroup frequencies [43] (Supplementary Tables S3 and S4; Figure 4A), show higher values for the Y-chromosome than mtDNA among the Bribri, Ngäbe, Emberá, Mestizo, and general population, which all bear some non-Indigenous male lineages (in different proportions). The opposite trend is observed for the Moreno and Guna groups, which present higher heterogeneity for mtDNA. The Naso show similar values for the two. This observation is also confirmed by the comparison of intra-population pairwise uniparental genetic distances (a measure of genetic diversity, Figure 4B): a much higher median value on the mtDNA gene pool is recorded for Bribri, Guna, and Moreno, thus testifying to a more diversified mtDNA gene pool with respect to the Y-chromosome one. Higher heterogeneity of the mtDNA pool is in accordance with the hypothesis of a very ancient IAm mtDNA legacy [36]. The high median values of the Y-chromosome heterogeneity estimates among the Mestizo may be due to the various allochthonous paternal contributions (both Western Eurasian and sub-Saharan African) in post-contact times, although this pattern does not appear in the general population.

**Figure 5:**
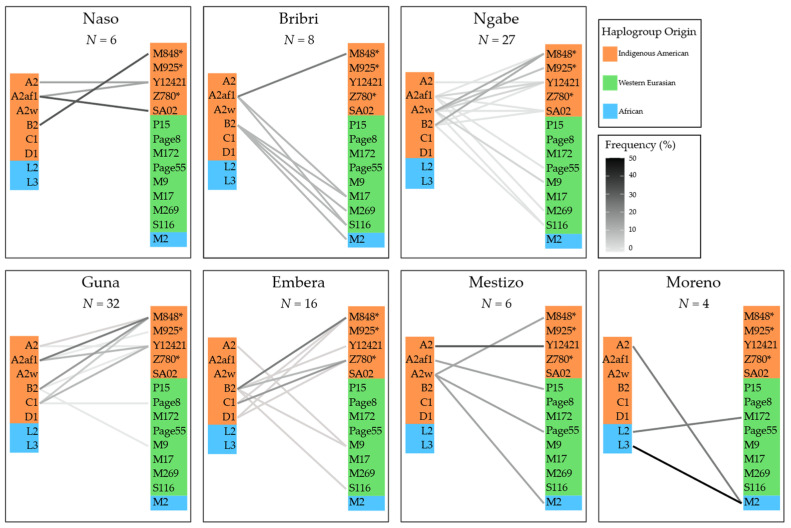
Connections between mtDNA and Y-chromosome haplogroups across Panamanian groups. Uniparental haplogroups found in Indigenous and mixed groups are listed in two columns (mtDNA haplogroups on the left, Y-chromosome on the right) and colored according to their geographical origin. Each line connects the maternal and paternal haplogroups of an individual; the intensity of the line is proportional to the frequency of that specific combination in the ethnic group.

The combinations of uniparental lineages that can be found in each individual from the different population groups are summarized in Figure 5. As expected, external contributions in the Indigenous groups mostly derive from WEu Y-chromosome haplogroups, with the mtDNA:Y-chromosome Indigenous lineage ratio ranging from 1:0.2 in the Bribri to 1:0.6 in the Guna and the Emberá. This is also confirmed for the mixed groups, with ratios of 1:0.4 in the Mestizo and 1:0.35 in the general population. The only notable exception is the Naso group where we find only IAm uniparental lineages (ratio 1:1), while the SAf source is predominant in the mtDNA pool of the Moreno group (1:0.75).

**Figure 6:**
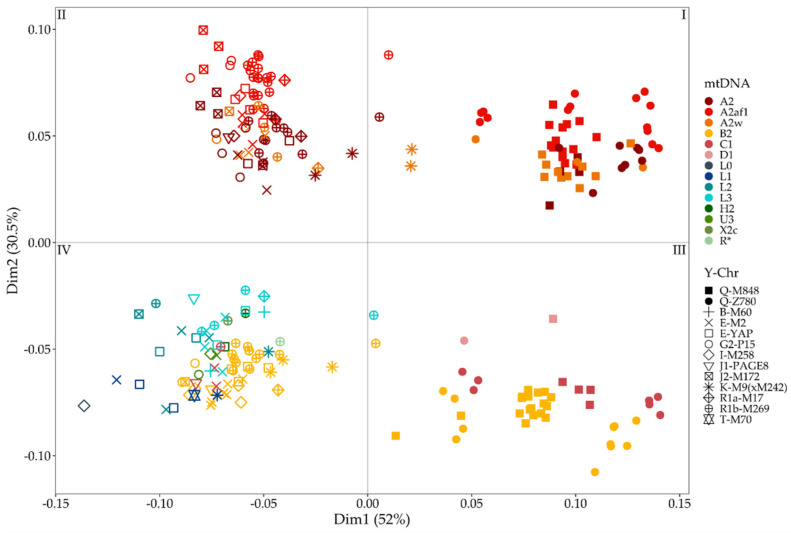
MDS plot computed on the mean of mtDNA and Y-chromosome pairwise distances.

To summarize the contribution of both uniparental lines to the gene pool of each individual, we took the mean of Y-chromosome and mtDNA pairwise distances and used the resulting dissimilarity matrix to compute an MDS (Figure 6). In the MDS, the first dimension separates individuals with both paternal and maternal Indigenous haplogroups; this separation is due to Y-chromosomes, while Indigenous mtDNA haplogroups form two main clusters along dimension two. The first cluster (quadrants I and II) includes only individuals belonging to A2, whereas the cluster in the third and fourth quadrants encloses all individuals belonging to the other haplogroups (B2, C1, and D1) together with individuals bearing European or African lineages (quadrant IV). These findings indicate that the Indigenous uniparental lineages largely contributed to the current genetic structure in Panama and further highlights the strong footprint of the mitochondrial lineages A2af1 and A2w. The same analysis has been performed on each population group, and the representativeness of the first two dimensions has been checked and confirmed (Supplementary Figures S2 and S3).

Taking into account the discriminatory power of haplogroups, we performed PCA analyses on both uniparental systems highlighting the separation of the Panamanian groups as a result of different histories in pre- and post-contact times.

**Figure 7:**
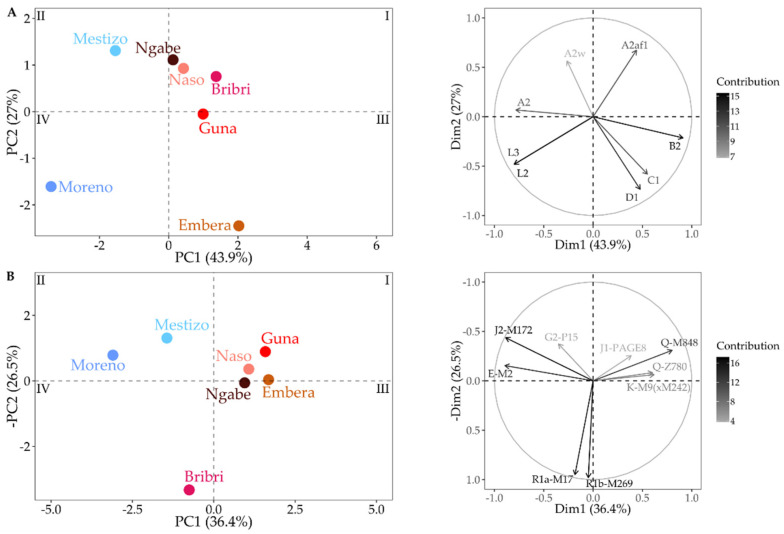
PCA computed on haplogroup frequencies of mtDNA (**A**) and Y-chromosome (**B**). The graphs on the right represent the variable correlation plots, which are colored according to the variables’ contribution to PC1 and PC2.

As for the Indigenous groups, the mtDNA confirms the greater genetic proximity of the western Panamanian groups (Figure 7A and Figure S4A), particularly between Naso and Bribri, as already identified in genome-wide analyses [36] and here further confirmed by the Correspondence Analysis (CA, Figure S4B). These features are not confirmed on the paternal side (Figure 7B and Figure S4C,D), which is mainly due to the high incidence of WEu lineages in the Bribri group, which are comparable to the general population. Both uniparental systems confirm the distinctiveness of the Moreno due to post-contact SAf haplogroups, while the Mestizo lie in an intermediate position due to the admixture of pre- and post-contact lineages in their gene pool. It is worth mentioning that although mtDNA lineages of Mestizos are all pre-contact, they are typical of different Indigenous groups.

## 4. Discussion

Previous studies have shown greater Indigenous maternal legacy [27] in the general population of Panama and a much lower Indigenous paternal component [28], which is mostly characterized by Y-chromosome lineages of Western Eurasian origin. This sex bias has been interpreted as a result of asymmetric coupling between male newcomers and Indigenous American as well as African women. A related hypothesis suggests that this sex bias could have been caused by more Indigenous men than women perishing or being deprived of reproductive rights after contact. This imbalance has been attributed to warfare and forced displacements of enslaved Indigenous males, which consolidated the female bias of post-contact Indigenous American survival. Another peculiar feature of these previous studies concerned the general population of Panama presenting a lower African male than female genetic legacy, which contrasts with the forced exportation of approximately two men for every woman in the trans-Atlantic slave trade [30,31] and may be related to subsequent social and cultural preferences [17,29].

In order to verify if and to what extent these genetic peculiarities characterize different population groups living in Panama today, we analyzed the mtDNA and Y-chromosome variation of a new sample collection, in which the individuals were grouped based on self-declared ethnic affiliations. These analyses confirmed a sex bias in the general population, which is characterized by high frequencies of Indigenous lineages for mtDNA (75.7%) and much lower for the Y-chromosome (13.4%). This trend is confirmed in other population contexts around the Isthmus, such as Mexico [40,63,64,65] and Colombia [24], with the extant admixed population showing mostly Indigenous mtDNAs (>90%) combined with European Y chromosomes (between ≈50% and 94%). However, this is not proved for other admixed populations. In Brazil, a predominant European origin is confirmed for the Y-chromosomes, but only ≈33% of mtDNAs on average (with local fluctuations) belong to Indigenous lineages [66]. Different yet not mutually exclusive hypotheses could explain these different patterns, including unbalanced or statistically inadequate datasets and the low resolution of uniparental screening of only modern individuals, but certainly, they testify to the complexity of admixture processes between populations with various cultural and biological backgrounds. An additional proof of this complex scenario is the different patterns detected in the Indigenous groups currently living in Panama: Indigenous mtDNA lineages reach 100% in all Indigenous groups, while the Y-chromosome haplogroups range from 25% in the Bribri to 100% in the Naso. Similar patterns, with a modest impact of allochthonous lineages on the maternal line (<5%) and paternal lineages from external sources present in varying proportions, have been detected in Indigenous groups currently living both to the north (Costa Rica, Nicaragua, and Mexico) [21,63,65] and to the south (Colombia) [26] of Panama, as well as in other regions [67]. Within the Isthmus, we pointed out a differential post-contact impact of allochthonous Y-chromosome lineages on the Indigenous genomic pool of the western Panamanian groups that were the most homogeneous in pre-Hispanic times [36]. In addition to a potential sample bias, particularly for the groups represented by less than ten individuals in our Y-chromosome dataset (Bribri and Naso), the alternative hypothesis based on the cultural system of the different population groups should be taken into account. Therefore, it is possible that Bribri women intermarried or otherwise coupled with men of both Western Eurasian and sub-Saharan African origins, and the newborns were considered members regardless of their paternal origin. Notably, the Ngäbe show admixture only with the WEu counterpart. Indigenous females and males of different origins formed part of the genetic heritage in the Mestizo and Moreno groups. It is noteworthy that we have re-evaluated estimates of African contributions to the general population when comparing the mtDNA and Y chromosome. The same values for both uniparental markers (16.8%) appear somewhat closer to historical data. These estimates are probably more accurate than those obtained in previous studies [27,28] where the self-declared ethnic affiliation was not recorded during sampling. Therefore, it is likely that some individuals of the general population were actually members of the Indigenous communities, where the incidence of sub-Saharan Y-chromosome lineages is much lower (≈1%; Supplementary Table S4).

Another outcome of the present work is the similarity between the two systems with respect to the significant difference in the haplogroup distribution among the Panamanian Indigenous groups (*p*-value < 0.001 for both systems). Indeed, mtDNA haplogroup A2w, previously found in North and Central American modern individuals [36,68,69,70,71,72,73] and more recently in an ancient pre-Hispanic individual excavated in Panama City [36], is only present in the Ngäbe group of our present sample. Likewise, in our sample, the male paragroup Q-M925* is exclusively found (except for one Guna individual) in the Ngäbe. The mtDNA haplogroups C1 and D1 are found in Emberá and Guna (only C1) in the east and not observed in western groups. Similarly, Q-Z780 Y-chromosomes from the west (Naso and Ngäbe) all belong to the sub-lineage Q-SA02, while those in the east (Emberá) remain classified as Q-Z780*.

Among the most frequent Panamanian sub-lineages are the mtDNA haplogroup A2af1 and the Y-chromosome paragroup Q-M848*. A2af1 has been mostly found in the Isthmo-Colombian area, including pre-Hispanic individuals [27,36,72], and in a few individuals from Central Mexico [40], aligning with a probable origin from the north. On the other hand, Q-M848* shows the same pattern of the mtDNA lineage A2af1, with high frequencies across all Panamanian Indigenous populations, especially in the Guna group (59.4% and 52.0%, respectively). This hints at the existence of an Isthmo-specific male source yet to be identified, which could mirror the A2af1 maternal side, indicating the Guna group as the most representative of a specific Pleistocene ancestry identified in the Isthmo-Colombian area (UPopI). Age estimates for A2af1 (15.82 ± 4.09 kya, [36]) and node Q-M848 (14.78 ± 0.02 kya; Unpublished data) are in line with this hypothesis. The low level of Guna Y-chromosome heterogeneity is probably due to a strong bottleneck in the population size before contact and/or loss of male lineages in post-contact times.

## 5. Conclusions

In summary, it is clear that both pre- and post-contact events contributed to shaping the uniparental gene pool of modern Panamanians. In pre-Hispanic times, the Indigenous groups probably developed in relative isolation from each other, forming three main clusters. A western Isthmian cluster encompasses Bribri, Naso, and Ngäbe, in spite of some dissimilarities in the latter (i.e., the mitochondrial A2w and the Y-chromosome Q-M925*). This cluster is only clear on the maternal side because of the matriarchal organization of many Indigenous groups, which leaves open the possibility of male introgressions of non-Indigenous lineages. In the eastern Isthmus, the Guna, who preserve the greatest traces of the newly defined autosomal ancestry UPopI, show a major legacy of very ancient uniparental footprints, the previously defined mtDNA haplogroup A2af1, and the Y-chromosome paragroup Q-M848*, while the Emberá show traces of inputs from the south (e.g., the mtDNA haplogroup D1). These different footprints are still evident mostly in the mitochondrial gene pool because the matrilineal lines of Indigenous populations were only marginally involved in post-contact admixtures.

A cultural implication of our findings is that the individuals who participated in this study declared themselves as belonging to Indigenous groups with reference to both sides of their genealogies, unaware of the non-Indigenous male components among their ancestors and confirming their matrilineal identifications. As for the post-contact input, we have confirmed a sex bias introgression of only paternal non-Indigenous lineages into local communities, but our findings also updated previous assessments concerning sub-Saharan African genetic input in post-contact times. Here, our new estimates regarding the general population, defined more accurately as individuals not identifying as members of any specific population group, contribute to solving an apparent discrepancy between genetic and historical data. However, this and other issues should be further investigated and directly tested through the analyses of (admixed) ancient genomes from colonial times. The importance of the ancient DNA analysis was already proved by our archaeogenomic analysis on the Isthmus of Panama [36], as well as in many other Indigenous population contexts [74] and references within. Therefore, our inferences on sex-biased admixture, here highlighted by the haplogroup sources of uniparental systems in present-day Panamanian groups, should be verified through a diachronic comparison with pre- and post-contact ancient individuals, in order to provide also a more accurate reconstruction of the demographic fluctuation over time in the Isthmian area. 

## Data Availability

All novel 431 mtDNA control-region sequences have been deposited in GenBank under accession numbers: OL344098–OL344528.

## Supplementary Materials

The following are available online at https://www.mdpi.com/article/10.3390/genes12121921/s1, Figure S1: Map showing the haplogroup distribution of the 248 mtDNAs according to the self-reported affiliation of the participants in the study (see Figure 1 for further details); Figure S2: MDS plots computed on the mean of mtDNA and Y-chromosome pairwise distances. The graph is divided by populations, and the total dataset is shown in the bottom right corner; Figure S3: (A) Elbow plot showing the eigenvalue for each dimension. The typical elbow is seen starting from the third dimensions, which implies that even two dimensions are enough to explain the variability in the data. (B) Shepard diagram showing the goodness of fit for the multidimensional scaling. The correlation was computed using the Spearman’s correlation test and Spearman’s rho and the associated p-value are reported on the plot; Figure S4: PCA and Symmetric Correspondence Analysis (CA) computed on frequencies of mtDNA (A and B) and Y-chromosome (C and D) haplogroups. Colored dots represent the populations, while gray arrows show the variables’ (haplogroup) contributions. Table S1: Panamanian individuals analyzed in this study; Table S2: Distribution of mtDNA haplogroups in the Panamanian populations analyzed. Frequencies (absolute and percentage) are also listed for the entire dataset (*N* = 431); Table S3: Distribution of mtDNA haplogroups in the Panamanian populations analyzed. Frequencies (absolute and percentage) are also listed for the entire male sample (*N* = 248); Table S4: Distribution of Y-chromosome haplogroups in the Panamanian populations analyzed. Frequencies (absolute and percentage) are also listed for the entire male sample (*N* = 248).
