# Supplementary material for: Weaving Mitochondrial DNA and Y-Chromosome Variation in the Panamanian Genetic Canvas
Source: Genes (Basel). 2021 Nov 29;12(12):1921. doi: 10.3390/genes12121921 (PMC8702192; doi:10.3390/genes12121921)
Supplement: Supplementary file 1 [file genes-12-01921-s001.zip › Supplementary Figures.pdf]

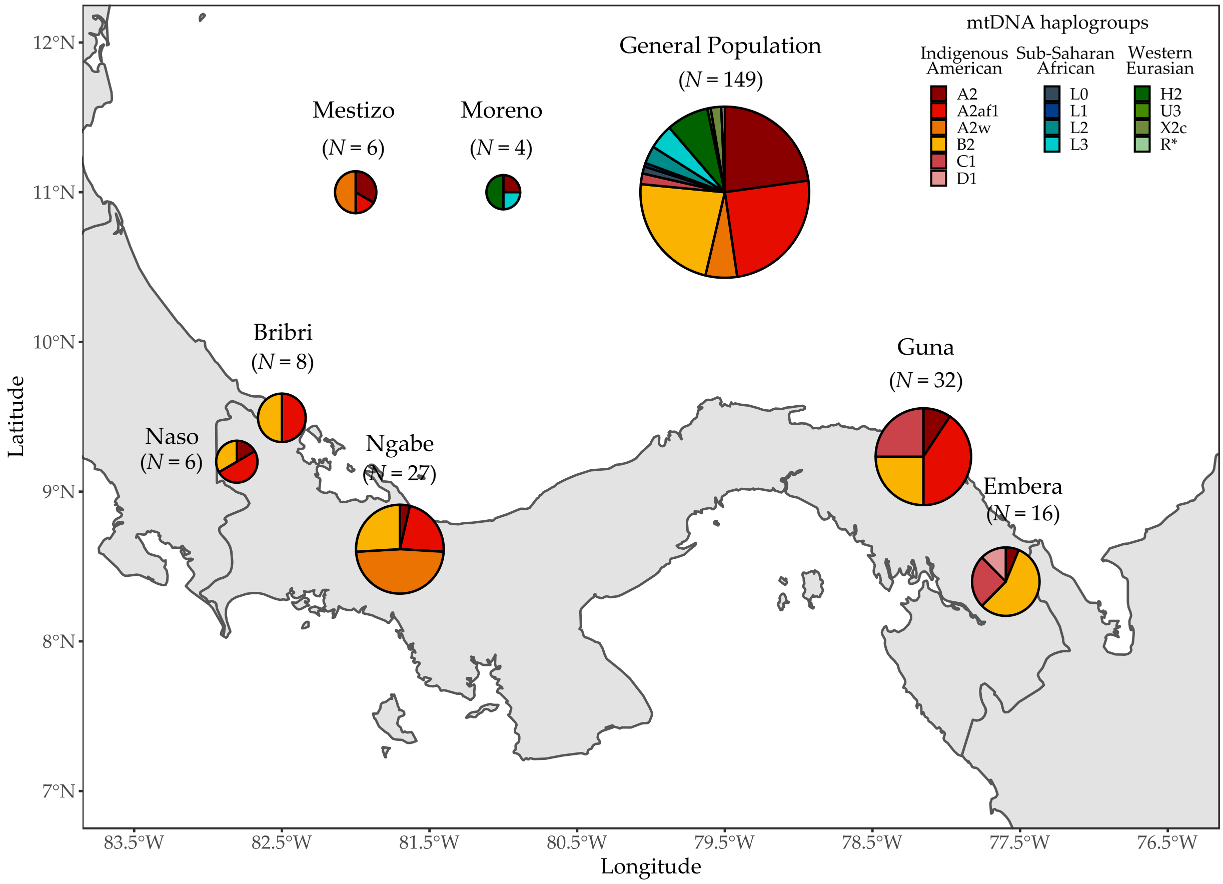

Supplementary Figure S1. Map showing the haplogroup distribution of the 248 mtDNAs according to the self-reported affiliation of the participants in the study (see Figure 1 for further details).

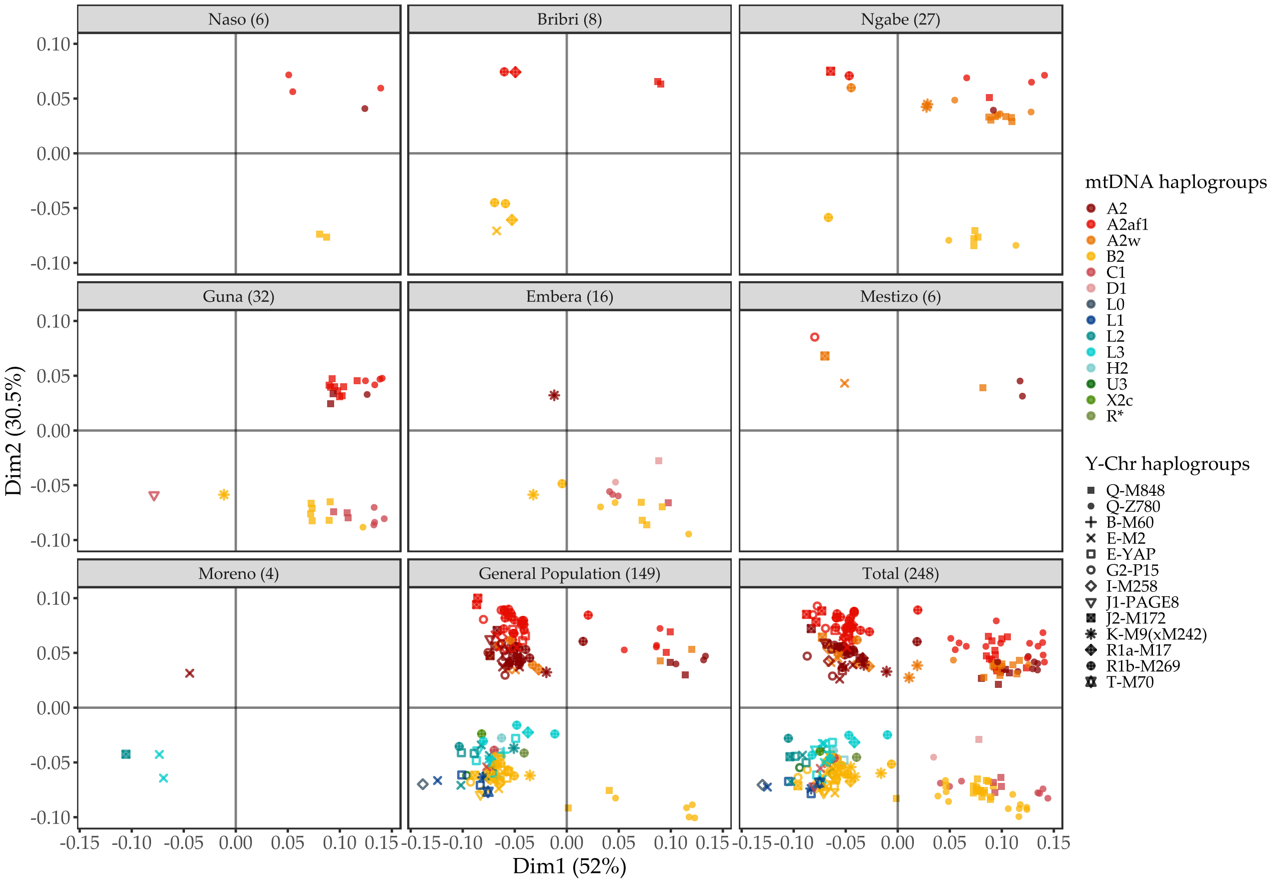

Supplementary Figure S2. MDS plots computed on the mean of mtDNA and Y-chromosome pairwise distances. The graph is divided by populations and the total dataset is shown in the bottom right corner.

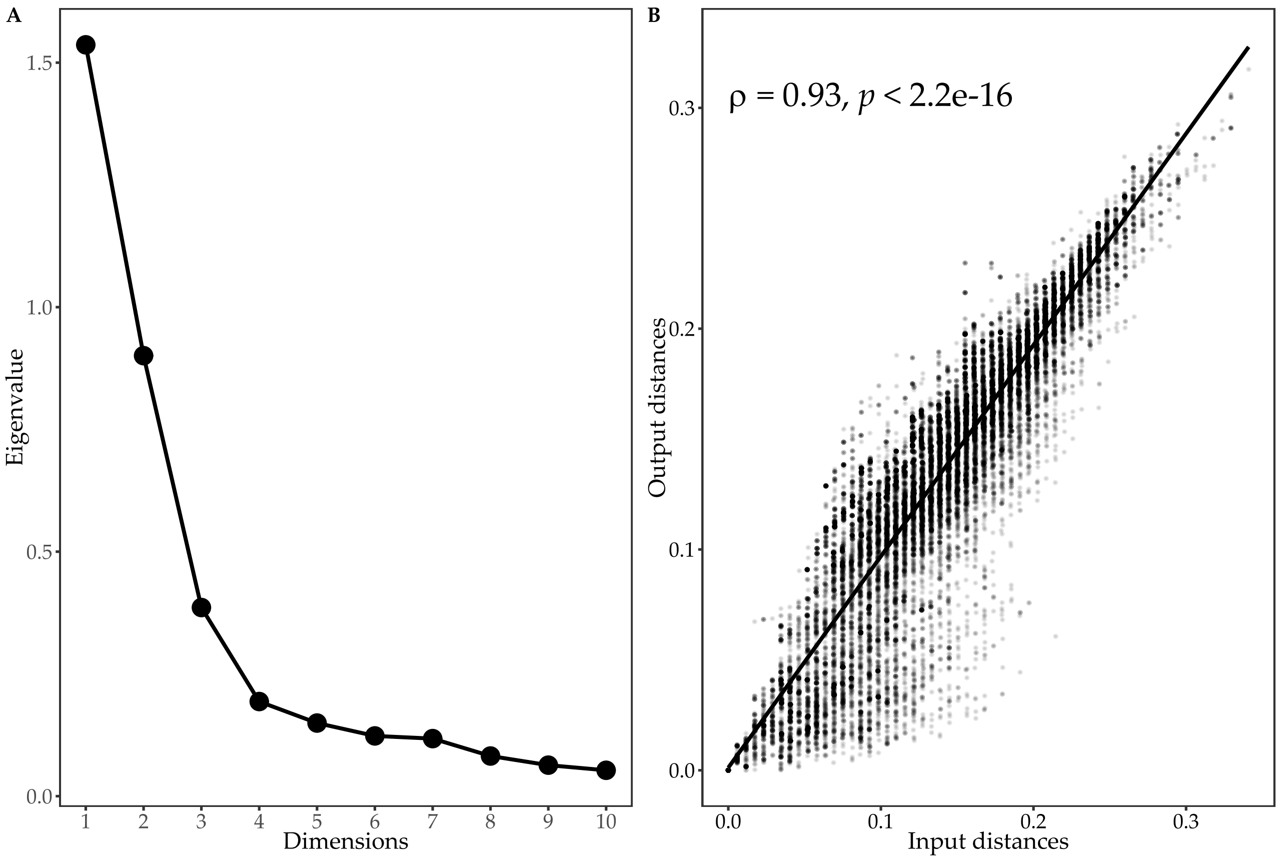

Supplementary Figure S3. A) Elbow plot showing the eigenvalue for each dimension. The typical elbow is seen starting from the third dimension, which implies that even two dimensions are enough to explain the variability in the data. B) Shepard diagram showing the goodness of fit for the Multidimensional scaling. The correlation was computed using the Spearman's correlation test and Spearman's rho and the associated p-value are reported on the plot.

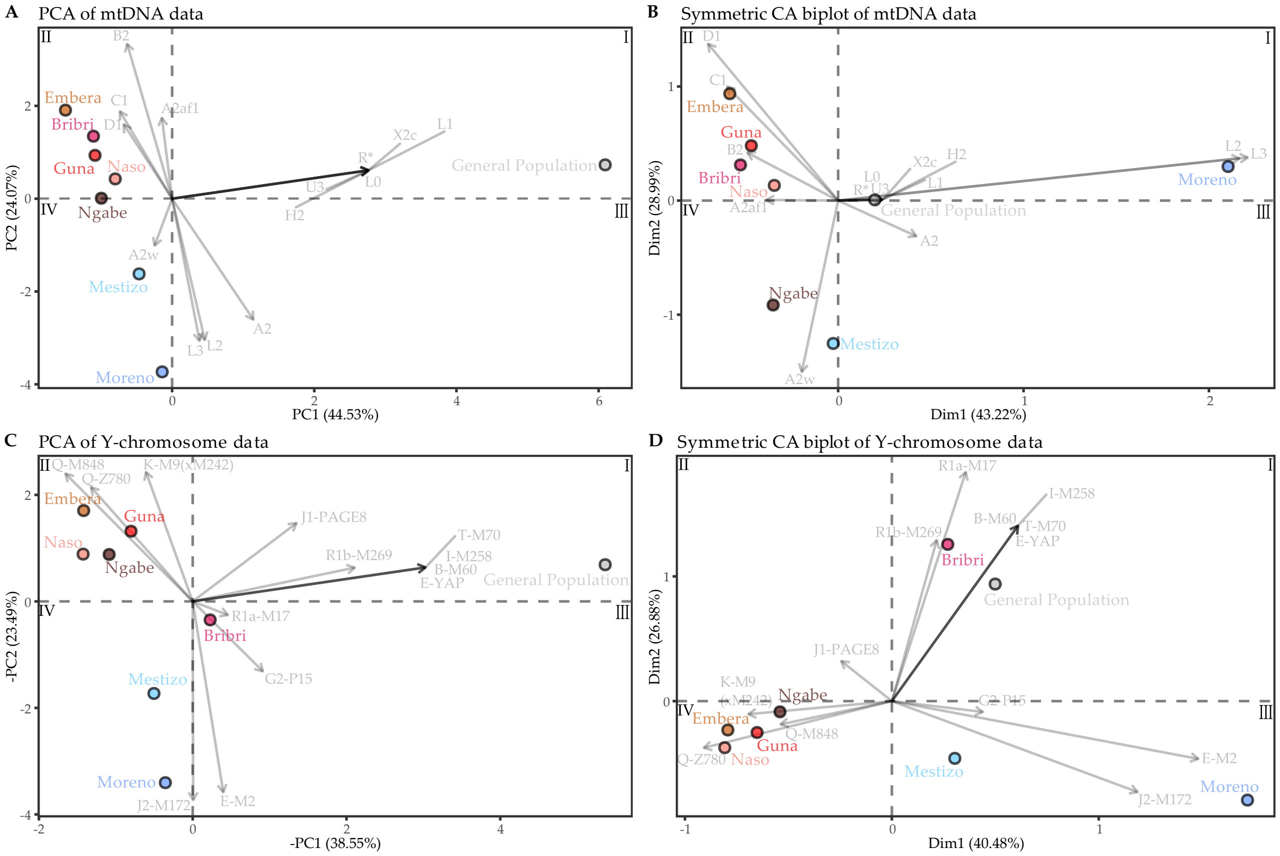

Supplementary Figure S4. PCA and Symmetric Correspondence Analysis (CA) computed on frequencies of mtDNA (A and B) and Y-chromosome (C and D) haplogroups. Colored dots represent the populations, while gray arrows show the variables' (haplogroup) contributions.
